# Neuropathology of the Brainstem to Mechanistically Understand and to Treat Alzheimer’s Disease

**DOI:** 10.3390/jcm10081555

**Published:** 2021-04-07

**Authors:** Ágoston Patthy, János Murai, János Hanics, Anna Pintér, Péter Zahola, Tomas G. M. Hökfelt, Tibor Harkany, Alán Alpár

**Affiliations:** 1 Department of Anatomy, Semmelweis University, H-1094 Budapest, Hungary; patthy.agoston@gmail.com (Á.P.); Janos.Murai@web.de (J.M.); hanics.janos@med.semmelweis-univ.hu (J.H.); panka.pinter@gmail.com (A.P.); zahola.peter@med.semmelweis-univ.hu (P.Z.); 2 SE NAP Research Group of Experimental Neuroanatomy and Developmental Biology, Hungarian Academy of Sciences, H-1094 Budapest, Hungary; 3 Department of Neuroscience, Biomedicum 7D, Karolinska Institutet, 17165 Stockholm, Sweden; tomas.hokfelt@ki.se (T.G.M.H.); tibor.harkany@ki.se (T.H.); 4 Center for Brain Research, Department of Molecular Neurosciences, Medical University of Vienna, 1090 Vienna, Austria

**Keywords:** serotonin, norepinephrine, noradrenaline, dopamine, neurodegeneration

## Abstract

Alzheimer’s disease (AD) is a devastating neurodegenerative disorder as yet without effective therapy. Symptoms of this disorder typically reflect cortical malfunction with local neurohistopathology, which biased investigators to search for focal triggers and molecular mechanisms. Cortex, however, receives massive afferents from caudal brain structures, which do not only convey specific information but powerfully tune ensemble activity. Moreover, there is evidence that the start of AD is subcortical. The brainstem harbors monoamine systems, which establish a dense innervation in both allo- and neocortex. Monoaminergic synapses can co-release neuropeptides either by precisely terminating on cortical neurons or, when being “*en passant*”, can instigate local volume transmission. Especially due to its early damage, malfunction of the ascending monoaminergic system emerges as an early sign and possible trigger of AD. This review summarizes the involvement and cascaded impairment of brainstem monoaminergic neurons in AD and discusses cellular mechanisms that lead to their dysfunction. We highlight the significance and therapeutic challenges of transmitter co-release in ascending activating system, describe the role and changes of local connections and distant afferents of brainstem nuclei in AD, and summon the rapidly increasing diagnostic window during the last few years.

## 1. Introduction

According to the report of Alzheimer’s Disease International (The World Alzheimer Report, 2019) over 50 million people live with dementia globally, a figure set to increase to over 150 million during the next 30 years (https://www.alzint.org/u/WorldAlzheimerReport2019.pdf accessed on September 2019). Presently, the annual cost of dementia care is estimated at US$1 trillion, and will be doubled with a sharp increase by 2030. Alzheimer’s disease (AD), a progressive illness with currently no effective treatment, accounts for two-third of dementia cases. This said, pioneering studies found that cognitive loss parallels histopathological signs: the accumulation of β-amyloid (Aβ) (1–40) and (1–42) extracellularly [1] and neurofibrillary tangles (NFTs) composed of hyperphosphorylated tau intracellularly [2], allowing cortical staging of the pathological process [3]. Obviously, it is cortical malfunction, which directly triggers and aggravates dementia. Accordingly, a wealth of studies has focused on neuropathological changes that progressively unfold in both allo- and neocortex. Thus, the bulk of work to date described phenotypic alterations in neuronal morphology and quantified synaptic loss in the cortex of humans and of relevant rodent models [4]. Clues to the etiology of AD include the Aβ cascade hypothesis [5,6], culminating, through malfunction in calcium homeostasis, in DNA damage, inflammation, dysregulation of energy metabolism, resulting in neuronal death and synapse elimination [7]. Parallel histopathology includes poor cytoskeletal instability which may be the consequence of, or might precede extracellular amyloid deposition and leads to the hyperphosphorylation of the tau protein [8,9]. Nevertheless, researchers could not hitherto identify the triggers, which launch the above cellular and biochemical changes. Instead, a kaleidoscope of molecular agents and candidate mechanisms emerged that might initiate and/or promote AD progression.

Amongst them, mitochondrial pathology and consecutive neurotransmitter dysfunction attracts growing interest. Impaired energy production can develop on the ground of oxidative stress triggered mtDNA mutations [10] or of disturbed calcium homeostasis due to disturbed calcium signaling [11]. This is paralleled by pathological morphology and axonal transport of mitochondria [12], which is further aggravated by the reduced number of autophagosomes and the increased dysfunctionality of lysosomes due to Aβ and hyperphosphorylated tau evoked PINK1 and parkin inhibition [13]. Further, impairment of mitochondrial proteostasis overloads the ubiquitin-proteasome system which leads to increasing intracellular Aβ load [14]. A major—clinical empirical—aspect that links mitochondrial pathology and neurotransmission malfunction is the effectivity of monoamine oxidase (MAO) inhibitors in AD; MAOs are present in the outer membrane of especially monoaminergic neurons, where they catalyze the breakdown of transmitters. Increased MAO activity leads to monoaminergic deficiency and mitochondrial dysfunction at presynaptic terminals through peroxidative stress [15].

Probably due to the cortical nature of AD, local factors and cellular actions were in the forefront of AD research and considerably less effort was taken to investigate the histopathology and mechanistic involvement of brainstem nuclei in AD. In this review, we summarize recent knowledge on how the brainstem is remodeled in AD, including diagnostic and treatment opportunities.

Brainstem houses nuclear groups of monoaminergic neurons with diverse projections and functions. Whilst their efferents towards the spinal cord typically act as supraspinal premotor command neurons to regulate vegetative function, their cortically-directed projections fine-tune cortical activity. This enables cortical function adaptation to different—outer and inner—environmental circumstances and challenges. Imbalance or malfunction in this tuning mechanism may not only transiently disturb homeostatic functions but lead to neurodegeneration. The consequent functional loss may not only imbalance in vegetative functions, mood, behavior, or sleep but also neuropsychiatric disorders [15]. Therefore, early diagnosis and therapeutic windows targeting brainstem biogenic amine centers offer a promising alternative in the understanding and treatment of AD.

## 2. The Serotonin System

Serotonin is an indoleamine produced from L-tryptophan with tryptophan hydroxylase 2 being the rate-limiting enzyme in its biosynthesis in the brain [16,17]. Like other monoamines, serotonin is metabolized by MAO, however, not by its A- (MAO-A), but by its B-type (MAO-B), which helps to eliminate intracellular competition of serotonin with dopamine or other monoamines at low concentrations [18]. Nevertheless, MAO activity is a significant source of reactive oxygen species, as well as ammonia [19], which make serotonin neurons vulnerable and MAO-B inhibitors a promising target in AD therapy [20]. The action of serotonin is terminated by reuptake via the serotonin transporter [21,22], which has remained one of the best therapeutic target in mood disorder therapy ever since [23,24].

The effect of serotonin, even within single neuronal circuits, is based on its triple-hit mode of action: in addition to its canonical synaptic and alternative paracrine release (volume transmission) [25] serotonin may produce hormonal effects via the blood stream [26]. At the same time, serotonin’s effect does not only depend on its three modes of action, but also upon the complex versatility of its receptors (http://journals.ed.ac.uk/gtopdb-cite/article/view/3155/4086 accessed on 16 September 2019). Actually, serotonin signals through seven distinct receptors (5-HT_1_–5-HT_7_), each with several subtypes which are different in terms of localization and downstream signaling [27]. Thus, most serotonin receptors are postsynaptic, yet 5-HT_1B_ and 5-HT_1D_ types act presynaptically, and 5-HT_1A_ can be found both in the pre- and postsynapse [27]. Mechanistically, 5-HT_3_ receptors are ion channels, hence having a direct influence on the cell processes. The other serotonin receptors are coupled to G-proteins: 5-HT_1_ and 5-HT_5_ inhibit, 5-HT_4_, 5-HT_6_, and 5-HT_7_ receptors activate adenylyl cyclase, respectively, whereas 5-HT_2_ receptors activate the phospholipase C cascade [27].

Throughout the brainstem, serotonin-containing^(+)^ neurons are clustered in cell groups, called raphe nuclei or referred to as B1-B9 cell groups [28,29]. The raphe nuclei are not solely composed of serotonergic cells, and, in humans, the majority (>80% of all neurons) of the cranial, but not of the caudal (10–20%) groups, express serotonin [30,31]. Cranial cell clusters (B5–B9), situated in the dorsal and median raphe nuclei, give rise to ascending projections to the frontal telencephalon, which is established in utero (i.e., embryonic day 18 in rodents) [32]. Fibers arising from the dorsal and median raphe nuclei are termed D- or M-fibers, respectively, which possess morphologically different types of termini in the cortex [33]. M-fibers typically form classical synaptic connections. In turn, D-fiber terminals lack a postsynaptic partner and operate via volume transmission [26]. The allocortex and subcortical brain regions show selectivity in which type of D- versus M-type termini they contain. The neocortex, however, receives both types of serotonergic innervation. Humans, as compared to other mammals, including primates, display an especially high density of serotonergic terminals in the infragranular layer of the prefrontal cortex [34], which are responsible for high-order behavioral organization and essential to working memory [35].

Serotonin emerges as a regulatory molecule already during brain ontogenesis to shape migration and axonal outgrowth [36], and an optimal expression level is critical for the normal development of the neocortex [37]. Early life serotonin dysregulation affects neural circuit formation and maturation. Impairment of serotonin signaling leads to different behavioral and social deficits, including mood and anxiety disorders [38]. This disease pathology finds its root in dysfunctional developmental plasticity, which can be shown by structural magnetic resonance imaging and reversed by selective serotonin re-uptake inhibitor treatment [39]. In adults, in addition to the wide range of its cardiovascular, gastrointestinal, endocrine, and other peripheral effects [40], serotonin affects diverse functions in the central nervous system. They include sensory perception, regulation of aggressive behavior, appetite, sex, sleep, mood, cognition, and memory related to the different targets and receptors of serotonin pathways [31]. Therefore, the impairment of serotonin function can be causal to vomiting and nausea, anxiety, depression, increased aggression, migraine, memory impairment, schizophrenia, dementia, sleep disorders, or mood imbalances [41].

### Pathophysiology

The argument that raphe neuron loss may trigger AD is supported by the fact that cortical serotonin degeneration precedes and parallels the course of memory deficits better than cortical Aβ deposition [42]. Especially regions of the default mode network, positioned at the medial convexity of our brain, suffer early serotonergic afferent loss and associated with consecutive cognitive impairments. Indeed, resting-state functional magnetic resonance imaging showed that serotonin transporter availability in the dorsal raphe nucleus and in the precuneus, i.e., origin and target of the serotonin system, respectively, decreases in early AD and parallels the altered resting state connectivity of the precuneus within the default mode network [42]. Magnetic resonance imaging of radiotracers has shown that reduction in levels of the serotonin transporter, a selective marker for serotonin projection integrity, is greater in mild cognitive impairment than after grey matter atrophy and reduction in local blood flow. These findings argue for compromised serotonin innervation as the perhaps earliest known sign of dysfunction in AD [43].

Yet, non-cognitive and cognitive deficits can be hardly specified with certainty as AD pathophysiology, since non-AD type dementias and other non-age-related neuropsychiatric illnesses share similar symptoms [44]. This is particularly due to the identically compromised functional wiring of the M- or D-fiber systems, affecting mood/memory or personality changes, respectively [45,46]. Similarly, correlations between the dysfunction of raphe neurons and disrupted control of sleep-wakefulness in early AD is indicative but not mandatory [15]. The sleep-wake cycle is in fact regulated by several monoamines and fast neurotransmitters, which work in subtle clock networks [47,48,49]. “Sundowning”, which is the increased agitation or activity in the evening hours, appears to involve over 40% of AD patients [50]. Moreover, fragmented sleep during night with daytime naps [51] is typically attributed to the malfunction of the brainstem serotonin system [52]. Underlying cellular histopathology includes both the accumulation of Aβ in the brain stem and the synaptic release of Aβ from raphe serotonergic terminals [53].

Post-mortem histopathology evidently supports the involvement of serotonergic neuron loss in AD: neurofibrillary tangles and neuropil threads appear early in cranial B cell clusters innervating the neocortex [54] (actually earlier than in the cortex itself [55]), with AD-specific clinical neuropathology, but not aging alone, being critical for significant cytoskeletal lesions [56]. Yet, raphe cell loss alone—in contrast to neuron loss in the nucleus basalis [57] or in the locus coeruleus (LC) [58]—does not correlate with the grade of cognitive and emotional decline [59]. This suggests a compensation potential and plasticity of the serotonergic system in which surviving raphe neurons may successfully take over the function of those cells that are lost, presumably by terminal sprouting [59]. Further, in early stages of AD, consequent cortical serotonin fiber loss parallels increased serotonin turnover characterized by the relatively elevated concentration of 5-hydroxyindolacetic-acid (5-HIAA) [60]. Despite any compensation capacity, however, raphe neuron loss ultimately leads to robust serotonergic denervation of both archi- and neocortex [56,61], which is also reflected in reduced serotonin level in AD brains [62]. This loss may follow the projection pattern of the hippocampal formation [63], starting from the entorhinal cortex—receiving profuse serotonergic input -, and spreading through the dentate gyrus and Ammons’s horn towards the neocortex [64]. Finally, effects mediated by any type of innervation critically depend on the type and quantity of specific receptors. Post-mortem analysis of AD brains shows subtype specific loss of 5-HT_1a_ and 5-HT_2a_ serotonin receptors in the allo- [65] and neocortex [66], respectively; or of 5-HT_6_ in frontal and temporal cortices [67]. These results have subsequently been underpinned by functional imaging studies [68]. The functional role of serotonin in AD is reflected in the therapeutic effect of drugs which target 5-HT receptors. Actually, 5-HT ligands are typically used to improve mood, memory, and cognitive functions, however, now with increasing interest in their application in AD therapy [69]. Due to the diversity of 5-HT receptors, ligands for their different subtypes affect different mechanisms. Thus, postsynaptic 5-HT_1a_ antagonists facilitate glutamatergic and cholinergic transmission [70], presynaptic 5-HT_4_ agonists increase the release of acetylcholine and reduce Aβ load [71], whilst 5-HT_6_ antagonists modulate the cholinergic or glutamatergic systems through disinhibition of GABAergic neurons [72,73]. Of note, 5-HT_6_ receptors can be targeted by alternative ligands, like ∆9-tetrahydrocannabinol (THC), whose chronic administration induces a long-lasting activation of mechanistic target of rapamycin (mTOR) in juvenile prefrontal cortex (PFC), leading to cognitive deficits in adulthood, at least in mice [74].

**Figure 1:**
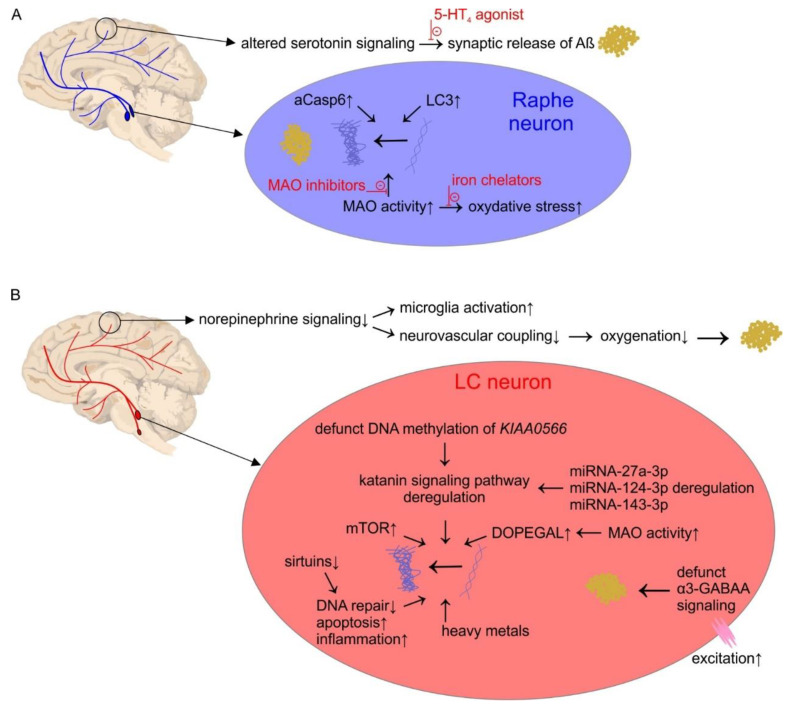
(**A**) Cortical and brain stem pathology of serotonin neurons in Alzheimer’s disease (AD). Agents written in red indicated therapeutic targets. (**B**) Cortical and brainstem pathology of norepinephrine neurons in AD. Abbreviations: DOPEGAL: 3,4-dihydroxyphenylglycolaldehyde, MAO: monoamine oxydase, LC: locus coerules, mTOR: mechanistic target of rapamycin.

Serotonergic raphe neurons and noradrenergic LC neurons show the earliest vulnerability to accumulated tau cytoskeletal pathology in AD before any cortical areas [55,75]. These neurons progressively degenerate with AD increasing severity, thus representing a crucial intervention target in early AD stages. Our understanding of the exact cell biological mechanisms that trigger AD is poor, but still, the few identified molecular pathological hallmarks are first identifiable in brainstem serotonin and LC neurons. Since neuronal loss and cognitive decline correlate best with the spread of NFTs [7], cellular pathways implicated in NFT formation or those connecting NFT neurotoxicity to neuronal dysfunction are critical in diagnosis and therapy (Figure 1A). As such, caspase-dependent and autophagy pathways have been repeatedly implicated in AD cellular pathobiology, since they shape the breakdown of misfolded or aggregated proteins in healthy cells [76]. This process becomes deficient in AD, which leads to intracellular accumulation of the toxic tau form, thereby contributing to cell death [76]. Although neuronal loss is only evident in Braak stage III (BB III), caspase and macroautophagy markers (aCasp6 and LC3, respectively) increase already from earliest AD stage (BB I) [55]. From the side of disturbed cellular metabolism MAOs attracted attention, since they produce toxic byproducts, including ammonia, aldehydes, and peroxides, when catalyzing monoamine oxidation [77]. Due to transmitter metabolism, the expression level of MAOs in brain tissue is high, especially monoaminergic neurons have huge MAO activity due to the continuous and large turnover of biogenic amines [78]. Enhanced, and an imbalance in, MAO activity triggers neurofibrillary tangle and Aβ aggregation; actually, MAO inhibitors emerged as potential drugs in AD treatment [79]. Along the same line, iron-chelators are also acknowledged as potential agents for neuroprotection in AD by reducing oxidative stress caused by increased MAO activity [80].

## 3. The Norepinephrine System

Norepinephrine is a catecholamine synthesized from dopamine by the enzyme dopamine β-hydroxylase and metabolized by MAO and catechol-O-methyltransferase (COMT). Norepinephrine can bind both to pre- and postsynaptic receptors, which are all of the metabotropic type [81], and its action is terminated by reuptake via the norepinephrine transporter [82].

Similarly to serotonin, norepinephrine exerts a dual synaptic/ non-synaptic action in both the periphery and the central nervous system [83]. Norepinephrinergic boutons diffusely innervate the cerebral cortex [84,85], where they contact non-neuronal elements, such as glial cells regulating local metabolism or inflammation [86,87,88] and blood vessels, thus shaping microcirculation [89,90,91]. Whilst postsynaptic α_1_ adrenoceptors are excitatory, α_2_ adrenoceptors inhibit neuronal firing perisomatically in a paracrine fashion [92] or presynaptically norepinephrine release [93]. Of note, both α_1_, α_2_ and β adrenoceptors include further subtypes, offering specific druggable targets. The origin of norepinephrinergic fibres resides in dorsal (A2, A4, A6) and ventral (A1, A5, A7) nuclear columns in the brainstem [28], with their majority arising from the pontine LC (A6) and with divergent multiple-target projection pattern also at a single-neuron level [94,95]. Norepinephrinergic afferents virtually innervate the entire central nervous system, including spinal cord, brainstem, diencephalon, telencephalic subpallium and the cortex itself [94,95]. Neurons of the LC excite the cerebral cortex principally through α_1_ receptor signaling [96,97]. In addition, however, LC neurons promote cortical wakefulness by exciting cholinergic and inhibiting GABAergic basal forebrain neurons, respectively [94], with GABAergic neurons projecting both onto the allocortex (hippocampus) and meso- and isocortex [98]. A further indirect cortex-exciting pathway originates from the norepinephrine system is through the activation of the serotonergic system via the cranial raphe nuclei [96,99].

### Pathophysiology

Its extensive efferent divergence posits the norepinephrine system to regulate many physiological processes [85]. Impaired norepinephrine signaling leads to an imbalance in autonomic, sleep/arousal, and photomodulatory networks, as well as to cognitive dysfunction. The loss of neurons in the LC is an early event in AD, perhaps even the very first one [75], which contributes to both brainstem- and cortex-related symptoms [15]. Thus, consistent with the roles of LC in the regulation of autonomic activity and arousal, both sympathetic activity and alertness decrease in AD patients, while the failure of the norepinephrinergic regulation of the oculomotor complex leads to malfunction of saccadic and smooth pursuit eye movements [100]. Of note, experimental reduction of norepinephrine in LC neurons triggers Aβ load in the prefrontal and temporal cortices in the rhesus monkey, likely suggesting an activity-dependency of amyloid production [101]. Obviously, cognitive decline caused by cortical failure is the most prominent sign of AD, both in its early [102] and later [103] stages.

Since LC is the only norepinephrinergic nucleus which innervates the cortex, its premature neuron loss contributes to early cortical dysfunction [100]. Both histopathological signs in the LC (extracellular amyloid deposition [104] and neurofibrillary abnormalities [102]) and clinical symptoms [105] clearly correlate with LC cell loss. Notably, norepinephrine innervation is especially dense in the hippocampal formation and the neighboring entorhinal cortex, where histopathological signs first appear and from where tau pathology spreads further to the neocortex. This fact could link early memory/cognitive dysfunction to the loss of LC afferents. Impaired non-neuronal modes of norepinephrine action also impel disease progression: diminished neurovascular coupling through astrocyte deficits leads to insufficient oxygenation [106], impaired blood brain barrier, and accumulation of extracellular amyloid [90]. Moreover, as an anti-inflammatory agent norepinephrine inhibits microglia activation via immunosuppression and the regulation of inflammatory genes transcription [107].

The cellular pathology of LC neurons, hence, potential triggers for AD onset, has been successfully addressed recently (Figure 1B). Heavy metals, like organic mercury, which are easily taken up by LC neurons are potential molecular triggers of AD cellular pathogenesis, i.e., induce tau pathology [108,109]. Toxin up-take in these neurons is further facilitated by their proximal position to the cerebrospinal fluid in the fourth of ventricle [110]. Microtubule-homeostasis of LC neurons is especially vulnerable: The katanin-signaling pathway for microtubule severing is deregulated in AD, particularly due to pathological DNA methylation of the Katanin-Interacting Protein gene (KIAA0566) [111]. Gene expression is also dysregulated at the post-translational level: Several microRNAs (miRNA-27a-3p, miRNA-124-3p, and miRNA-143-3p) are de-regulated in the LC already at the first stages of neurofibrillary tangle formation with an exact cellular mechanism yet to be explored [112]. Norepinephrinergic neurons show selective vulnerability towards metabolic challenges, too: 3,4-dihydroxyphenylglycolaldehyde (DOPEGAL), the side product in MAO-A metabolism of norepinephrine, triggers tau pathology in LC neurons and propagates its spreading [113]. Overall, the vulnerability of tau metabolism in norepinephrine cells offers druggable substrates, including proteins which critically influence cellular processes. The nicotinamide adenine dinucleotide (NAD^+^)-dependent deacylase sirtuins are longevity gene products, for example, involved in metabolic regulation, DNA repair, transcription, apoptosis, and inflammatory processes, the reduced expression of sirtuins in AD triggers, and its overexpression blocks, taupathy, respectively [114,115]. Conversely, reducing upregulated mTOR level—a typical enzymatic blueprint in neurons predicted to develop tau hyperphosphorylation—have beneficial effects on tau pathology [116]. Similarly, nitric oxide signaling and redox homeostasis failure is a dangerous foible of norepinephrine cells, which contributes to dysregulation of stress systems and exacerbates cognitive impairment [117]. Overexcitation of norepinephrine cells is a further mechanism which critically impairs norepinephrine release, influencing emotion and cognition. Intracellular Aβ oligomers are portent candidates in this process through altering α_3_-GABAA receptor expression in AD [118].

## 4. The Dopamine System

The third major brain stem biogenic amine transmitter is the catecholamine dopamine, which shares similar synthesis and metabolism to norepinephrine. Thus, it is synthetized from tyrosine by its rate limiting enzyme tyrosine hydroxylase followed by decarboxylation by amino acid decarboxylase (AADC), and is actually the precursor of norepinephrine. Dopamine acts through different receptors in human: D_1_-like receptors (D_1_ and D_5_) stimulate [119], whereas D_2_-like receptors (D_2_, D_3_, and D_4_) inhibit [120] adenylyl cyclase, respectively, by acting through G-proteins. In addition to its postsynaptic action involved in many functional systems, dopamine receptors occur presynaptically, which in case of D_1_ receptors not only promise druggable targets in psychosis or schizophrenia [121] but can elaborately tune GABAergic collateral inhibition in medium spiny neurons of the parkinsonian striatum [122]. Synaptic action of dopamine critically depends on the action of dopamine (and cocaine) transporter (DAT) which terminates neurotransmitter signal by removing dopamine from the synaptic cleft [123,124]. Therefore, DAT emerged as a valuable pharmacological target in the diagnosis and therapy of different neuropsychiatric disorders, including Parkinson’s disease [125], sleeping disorders, or addiction [126].

Dopamine is produced in four midbrain nuclei, termed A8–A11 [127]. Whilst serotonin and norepinephrine innervations promote cortical alertness and regulate vegetative functions, the dopamine system is involved in motor, memory/behavior, and reward control. The compact part of substantia nigra (A9) mainly projects to the dorsal striatum to shape voluntary motor activity, a pathway critically impaired in Parkinson’s disease [128,129]. Dopaminergic neurons of the ventral tegmental area (VTA, A10), in turn, project to the neocortex (mesocortical pathway), to the archicortex and nucleus accumbens (mesolimbic pathway), and to the cortical basolateral amygdala (mesoamygdaloid pathway) to adjust reward, working memory, and vigilance [130]. Early on, researchers did not relate any damage of the dopamine system to AD: Dopamine transporter sites, tyrosine hydroxylase (TH), and D_2_ autoreceptors were not changed in the substantia nigra, and ventral tegmental area of postmortem AD subjects, as well as loss of striatal dopamine transporter sites, occurred only in relation to clinical parkinsonian symptoms [131]. Similarly, the number of TH and DAT-expressing midbrain dopamine neurons and DAT mRNA expression of surviving cells significantly decrease in Parkinson’s disease, but not in AD [132]. In vivo striatal DAT imaging studies showed that dopaminergic presynaptic function is preserved in AD, and extrapyramidal features in AD are related to postsynaptic changes in the striatum [133]. This was later confirmed in the APPswe/PS1DeltaE9 transgenic AD mouse model where dopaminergic nigrostriatal dysfunction paralleled striatal Aβ load [134]; hence, dopamine neuron loss is likely due to postsynaptic amyloid toxicity instead of an innate and primary vulnerability of dopaminergic neurons.

In contradiction with the above, involvement of the dopamine system in AD was addressed and also identified already over thirty years ago when dopamine transporter reduction [135] and neurofibrillary tangles were reported in VTA neurons in AD [136]. Further, dopamine and its metabolites levels were reduced in several brain regions, including cortex [137]. Recently, VTA cell loss was reported in the Tg2576 AD transgenic mouse model which preceded amyloid load or neurofibrillary tangles development, but paralleled progressive dopamine outflow and impaired reward processing and memory disturbances [138]. In addition to dopamine and its metabolite reduction, dopamine receptor decline [61,139] may also contribute to AD pathogenesis, partly due to select DRD1 B2 and DRD3 1 allele polymorphism [140]. Similarly, neuropsychiatric symptoms due to the impairment of the limbic system in AD patients, which appear actually earlier than dementia [141], are likely associated to mesolimbic and mesoamygdaloid systems deficit [15]. Whilst a causative link is currently lacking, quantitative MRI showed that VTA disruption correlates with memory impairment in early AD [142]. In a large AD patient cohort, including patients with mild cognitive impairment and patients with AD dementia, as well as healthy individuals, early neuropsychiatric symptoms, including aggression, irritability, and sleep and eating disorders, were directly associated to VTA connectivity loss, primarily affecting the so-called default mode network [143,144]. Significant tissue and metabolic connectivity loss in the mesocortical, but not in the mesostriatal pathway, was shown using in vivo 3T MRI and 18-F-FDG-PET scans, which underpins the involvement of the mesocorticolimbic system in AD [145]. 

Recently, the caudate nucleus also emerges as a possible locus for specific AD symptoms: apathy, a common neuropsychiatric symptom in AD patients, correlates with dopamine transporter levels in the caudate nucleus [146]. In AD transgenic mice models, EEG modifications in (but not restricted to) 5xFAD mice were associated with alterations in dopaminergic transmission [147], and reduced dopaminergic innervation in the Tg2576 hippocampus resulted in reduced synaptic plasticity and excitability of dorsal subiculum pyramidal neurons [148].

The lack of causality between brainstem dopamine system failure and AD did not hinder pharmacologists to investigate the possibility of molecular dopamine therapy in AD, with efforts accelerating in the past five years. Inhibition of amyloidogenic or tau fragments aggregation was targeted by dopamine-based hybrid molecules [149], by the reactive catecholaldehyde intermediate of dopamine metabolism DOPAL [150], multifunctional dopamine D_2_/D_3_ receptor agonists [151], dopamine appended derivatives of naphthalenediimide (NDI) [152] and by other rationally designed structural derivatives based on dopamine’s oxidative transformation [153]. Promising pharmacological targets include glial inflammatory pathways: dopamine receptor D_3_ signaling in astrocytes promoted neuroinflammation [154], whereas dopamine D_1_ receptor agonists improved neuroinflammation in AD and consecutive Aβ1-42-induced cognitive impairment [155].

## 5. Co-Release of Biogenic Amines

Accumulating evidence supports that the actual action of synapses depend on the co-release of structurally different neurotransmitters and neuromodulators. A wealth of studies detailed the co-release of fast neurotransmitters, neuromodulators, and other peptides, reflecting activity-dependence or synapse maturation during ontogenesis (e.g., Reference [156,157,158]). Here, we mention aspects which are related to biogenic amines being typical examples of this operation which must alert us to critically estimate the role of brainstem projections and their released substances in AD.

Co-release from major monoamine brainstem efferents does not necessarily include a further substance, i.e., other than serotonin, norepinephrine, or dopamine. Although the canonical norepinephrine and dopamine pathways from the ventral tegmental area and the LC, respectively, have been previously described to maintain dissociate function, LC can simultaneously broadcast both substances in the brain [159]. Actually, in the medial prefrontal cortex, the primary source of dopamine are norepinephrinergic terminals [160]. Co-release of norepinephrine and dopamine in the archicortex is also critical: learning/memory tasks and novelty-induced memory consolidation rely on dopamine release from LC neurons in the hippocampus [161,162]. 

In addition to these major monoamine transmitters, A1–A7 and B1–B8 field neurons release further neuropeptides. Especially, noradrenergic neurons may contain and release neuromodulatory peptides other than noradrenaline [163,164,165,166]. Co-transmitters in peripheral (sympathetic) noradrenergic neurons include adenosine triphosphate (ATP) and neuropeptide Y (NPY) [163,167,168], and, in central norepinephrinergic neurons ATP [163], NPY [169], enkephalins [170], and galanin [169,171,172]. Also glutamate has been reported to be a co-transmitter in LC neurons [170,173], but others have identified the vesicular glutamate transporter type 2 (VGLUT2) in medullary epinephrinergic but not in norepinephrinergic neurons [174]. Of note, the co-transmitter may modulate the action of noradrenaline at both pre- and postsynaptic sites, regulating release or neurotransmission, respectively [163,167,175]. Cocaine- and amphetamine-related transcript peptide (CART) and brain-derived neurotrophic factor (BDNF) can be also released from LC neurons [173]; these substances emerge as useful targets in AD therapy due to their role in anti-inflammatory [176] and neurotrophic signaling [177], respectively.

Amongst these neuropeptides galanin attracted special attention. Galanin expressing norepinephrinergic fibers richly innervate the cortex and virtually all of these afferents in both neo- and archicortex arise from the LC [178,179]; serotoninergic raphe and cholinergic basal forebrain nuclei send fibers to these targets but galanin has so far not been detected in these afferents [180]. Galanin mainly acts through GAL1 and GAL2 type receptors [181], the latter also conveying neurotrophic actions [182]. GAL1 and GAL2 receptors are present in vertebrates as diverse as fish and primates, whereas GAL3 is expressed only in some mammals, including human [183]. The GAL3 type receptor may be relevant in the human brain, e.g., in LC neurons, but is limited in rodents [171,184]. Actually, the *GAL_3_* gene was implicated in alcohol addiction among two ethnically and geographically diverse human populations due to a single nucleotide polymorphism (SNP) [185]. The mesocorticolimbic effect of galanin does not solely depend on cortical efferents, though: LC fibers project to VTA neurons and involve an indirect mode of galanin signaling [181,186]. Thirdly, galanin may act in an autocrine manner with evolutionary distinctions: its dendritic and somatic release inhibit LC neurons through GAL1 receptors in rats [187] but probably less so in mice [188]. Galanin may play a role in depression-like behavior, including being involved in resilience mechanisms [172,189,190]. Its neurotrophic/neuroprotective and plasticity-promoting effect drew scientists’ attention to galanin’s role in neurodegeneration. Even if AD may be initiated in the LC, those neurons that express galanin are spared even in late stage AD [191]. Interestingly, galanin overexpression is believed to preserve forebrain cholinergic neuron function, which may in turn delay the onset of symptoms of AD [180]. Whilst an exact cellular machinery remains unexplored in vivo, in vitro results suggest involvement of inhibition of p53, Bax, and caspase-3 reducing neurotoxicity [192], galanin’s neuroprotective action, especially against Aβ toxicity [193,194,195], argues for its role to slow or reverse neurodegeneration [180]. Nevertheless, recent advances showed that galaninergic hyperinnervation was infrequent and particularly uncommon in advanced/severe AD, which suggests that higher burdens of co-existent AD pathology impair the highly inducible galaninergic response [196]. Developing subtype-selective galanin ligands offer novel druggable target in therapy which include neurodegenerative diseases [197].

One of the most abundant neuropeptides in the brain is neuropeptide Y (NPY), with a rich representation in cortex mainly expressed in GABAergic interneurons [198,199]. NPY acts via several receptors [200], which are widely distributed in the brain [201]. Exogenous infusion of NPY has robust effects: it particularly stimulates feeding behavior [202] and affects sleep/awake cycles [203]. However, no evidence of a physiological in vivo mechanisms involving brainstem-derived NPY has been hitherto identified. Neurons of norepinephrinergic and epinephrinergic nuclei of the ventrolateral medulla, as well as many LC neurons co-express NPY [169,179,204]. (Of note, mid- and forebrain dopamine neurons (A8–A15) do not express NPY [204].) These neurons innervate the subcortex, including the amygdala, where the LC-amygdala projection shapes inhibitory avoidance in rats [205]. Based on retrograde studies, fibers arising from norepinephrine/NPY LC neurons, however, reach both archi- and neocortex: the retrohippocampal region/entorhinal area [206,207] and most cortical regions without specific preference [207], respectively. Early studies reported that neocortical NPY decreases in AD [208], which indicated that NPY can be a promising target in the therapy of AD [209]. However, despite cortical fibers originating from brainstem norepinephrine centers, i.e., from norepinephrine/NPY LC neurons [206,207], no mechanistic link between LC-derived NPY and AD has been hitherto identified. Actually, there is no direct evidence that NPY peptide is exported in the efferents from LC [179]. Yet NPY is a most prioritized gene on convergent functional genomics ranking in AD [210]; it is neuroprotective and anti-inflammatory, exerts trophic support [211]; further, it protects cells against neurotoxic damage from Aβ peptides [212]. Therefore, this NPY may very well originate from cortical interneurons. In mechanistic detail, in vitro studies show that NPY does not only offset Aβ toxicity, e.g., through its C-terminal fragments cleaved by the γ extracellular endopeptidase neprilysin [213], but restores neurotrophin levels, at least in neuroblastoma cells [214]. A further NPY-triggered neuroprotective mechanism against Aβ toxicity includes the decrease in miR-30a-5p expression which increases brain derived neurotrophic factor (BDNF) mRNA and protein levels [215]. NPY reduces Ca^2+^ influx in presynaptic terminals, which protects neurons against Aβ-induced excitotoxicity by the inhibition of voltage dependent Ca^2+^ channel [216]. Through its Y2 and Y5 receptors, NPY activates protein kinase A and p38K, which inhibits necrosis and apoptosis [217].

## 6. Brainstem Monoaminergic Nuclei in AD: Interconnections and Afferent Loss

The balance between serotoninergic, norepinephrinergic, and dopaminergic release from ascending brainstem projections [218] diagrams a dynamic fine-tuning of brain activity and cortical alertness; however, local circuits emerge as important regulators in this function. LC and the dorsal raphe nucleus are mutually interconnected: norepinephrine act on raphe neurons via α_1_ receptors [219], and serotonin binds to 5-TH_2a_ receptors on LC neurons [220]. Noradrenergic nuclei directly project to the VTA [221], and the disruption between dopamine and norepinephrine brainstem centers in AD have been mapped in vivo [222]. In addition to altered innervation between brainstem monoamine centers in AD—like degeneration of cholinergic innervation of the LC [223]—, disturbed autocrine signaling may emerge as an alternate mechanism to trigger malfunction. This is not only realized by compromised serotonin [224], norepinephrine [225], or dopamine [226] auto-innervation but also through somatic or dendritic release and binding of neuropeptides, like galanin [187].

Altered non-brainstem afferents onto brainstem monoaminergic nuclei are probably critical contributors to impaired norepinephrine, serotonin and dopamine signaling. A plethora of different and functionally critical afferents reach brainstem monoaminergic neurons [15], ranging from lateral hypothalamic oxytocin innervation of LC and raphe neurons through different oxytocin receptors [227], to prefrontal afferents which provides tonic activation of the LC [228,229]. Nevertheless, only few of the known afferent signals have been directly and mechanistically addressed in, and could be related to AD. Amongst these, somatostatin innervation of LC neurons attracted special attention, since the density if somatostatin binding sites is very high in the LC [230] and somatostatin loss is an established hallmark of AD [231]. In addition to impaired direct cortical effects [232], defunct somatostatin signaling via somatostatin type 2 receptors (Sstr2) causes an elective, global and progressive noradrenergic axonal degeneration [233]. The role of brainstem afferents and its dysfunction in the neuromodulation of memory formation and extinction in AD is just beginning to unfold. Malfunction of engram circuits—dominated by the medial prefrontal cortex and hippocampus—massively depend on the interaction with select brainstem centers, including periaqueductal gray, LC and raphe nuclei, and their impaired signaling results in memory failure [234].

## 7. Other Brainstem Pathologies in AD

In addition to the monoaminergic nuclear systems which shape cortical function, brainstem harbors several centers which regulate visceral and reflex functions. Several of these centers can undergo early damage in AD, which accounts for typical but non-cortical AD-related symptomatic. A particular example is the visual system, which suffers early impairment to neuronal damage in several of its centers. Thus, neurofibrillary pathology in neurons of the tegmentopontine reticular nucleus (nucleus of Bechterew) appears already in preclinical AD, which results in abnormal generation of horizontal saccades and smooth pursuit movement of the eyes [235]. Rapid vertical eye movement is impaired because of cytoskeletal damage in the neurons of, and Aβ deposition in, rostral interstitial nucleus [236]. Moreover, altered, repetitive light reflex is due to Edinger-Westphal nucleus pathology reflected in dendritic and dendritic spine degeneration [237]. Visceral dysfunction is caused by the impairment of vegetative brainstem centers. So, nuclei of the pontine parabrachial region suffer similarly early involvement of cytoskeletal pathology, yet, due to their complex regulatory role, their exact contribution to AD symptomatic is difficult to identify [238]. Impairment of periaqueductal grey neurons may be responsible not only for sleep [47], but their altered connectivity contributes to altered pain perception in AD [239]. The efficient, high-resolution MT-weighted sequence at 7T successfully images the solitary tract nucleus [240], which enables us to monitor early changes in this essential complex signaling hub of visceral functions, hence vector in the upstream factor in AD [241]. Whereas several further brainstem nuclei can be associated with AD-related neuropathology [242], a direct causative link or contribution to AD remains ambiguous.

Hypertension typically occurs in AD patients, which can find its root in the impairment of relevant brainstem nuclei, the solitary tract nucleus and the epinephrinergic/norepinephrinergic ventrolateral medulla. This neurogenic form of hypertension is caused by the imbalance in the brain renin-angiotensin system medulla [243,244]. A hyperactive brain renin angiotensin system, oxidative stress, and consecutive neuroinflammation in brainstem catecholaminergic centers trigger hypertension and this effect occurs via angiotensin receptor mediated mechanisms [245]. Actually, centrally acting angiotensin receptor blockers in AD individuals improve cognitive functions [246]. Considering the strong correlation between cognitive decline and high blood pressure [247], hypertension can serve as a useful hallmark of neurological disorders, including AD [248].

## 8. Diagnostic Possibilities of Brainstem Pathology in AD

The brainstem undergoes early and progressive changes in AD. Detection of these changes could contribute to the early diagnosis of AD (even in the preclinical phase), widening the window for therapeutic intervention. Neuropathological evidence shows that 20% of Braak stage 0 and all Braak stages > 1 have neurofibrillary changes in the dorsal raphe nucleus [249], and 2.6% of dorsal raphe neurons and 7.9% of LC neurons have hyperphosporylated tau already in Braak stage 0 [55]. Further, the LC volume—but not the neuronal number—is reduced by 8.4% for each Braak stage advance so that the LC volume shrinks already by an average of 25% until characteristic AD symptoms and neuronal loss develop, i.e., in Braak stage 3 [250]. As we have seen, brainstem biogenic amine projections impact several functions and brain states, such as learning, memory, attention, mood, anxiety, and sleep-wakefulness, and their early loss accounts for the development of behavioral and psychological symptoms of dementia which occur months or even years before the onset of cognitive impairment [15].

Indirect, yet powerful diagnostic tools include pupillometry, liquor (CSF) and plasma norepinephrine measurements. These approaches are actually based on LC dysfunction. Since LC activation correlates with pupil dilation, normal individuals with high AD risk show increased task-evoked pupillary dilatation [251]. This can be explained by the presumable compensatory noradrenergic hyperactivity in early disease stages [252]. Increased CSF and plasma norepinephrine levels also reflect a higher LC tone in AD [253]. A recent study has shown that high versus low plasma norepinephrine levels—which correlate with Mini Mental State Examination (MMSE) score in AD patients—represent the early versus late stages of the disease, respectively [254]. These results mirror the early overactivation and subsequent regression of the LC in AD.

The advent of last generation medical imaging provided unprecedented resolution in AD diagnosis, especially since reliable early biomarkers are lacking. Medical imaging diagnostics not only proved the parallelism of LC pathology and clinical symptoms but also offered predictions for AD. Moreover, they can differentiate AD from normal aging where LC changes are lacking or minimal [255,256]. Since LC suffers early in AD with in vivo detectable neuronal loss [257,258,259], and is the origin of trans-axonal propagation of tau pathology, the examination of the LC—transentorhinal cortex pathway is especially critical. Thus, by leveraging the high-resolution and multi-shell diffusion MRI data from the Human Connectome Project, decreased fiber integrity was demonstrated in the above tract in correlation to increasing disease severity [260]. In late-onset AD patients, fMRI showed decreased whole-brain global connectivity with LC-cerebellum positively correlating with neurocognitive scores [261]. Presently, dynamic function of LC can be traced using ultra-high-field multi-modal 7T fMRI imaging [262], which offers a new scope for functional medical imaging in the near future. MRI characterization of neuromelanin—a pigmented polymer that results from the oxidation of catecholamines, including norepinephrine in LC neurons [263]—has emerged as a promising diagnostic tool [264], since its in vivo signal for melanin is intense and positively correlates with the cognitive reserve and verbal intelligence [265]. Besides these investigations, several studies argue for contrast/intensity ratio measurements at exact brainstem loci in AD diagnosis. Thus, section located 10 mm caudal to the left inferior colliculus has the highest potential in differentiating between healthy controls and AD patients [266], LC contrast was significantly lower in patients with AD, but not in subjects with mild cognitive impairment [258]. Further, LC signal intensity was lower in AD patients (regardless of typical and atypical presentation) from the prodromal stage, which was independent on amyloid load, but paralleled the episodic memory score [257].

In addition to targeting the LC by neuroimaging, several other structural brainstem MRI studies have been encouraging for achieving an improved AD diagnosis. Thus, AD patients show smaller brainstem volumes with significant deformations in its upper, posterior part, corresponding to the location of the raphe nuclei [267]. Voxel based morphometry has recently provided evidence that patients with mild AD show bilateral loss in the pons and in the left part of the midbrain as compared with normal controls [268]. The study of Dutt et al. seems particularly promising, as they examined cognitively normal individuals, who later progressed to AD dementia, and found smaller baseline midbrain volumes and revealed specific volumetric reduction of the LC [269]. This raises the possibility that brainstem volumetrics—normalized to total intracranial volume—could have a great value in predicting future progression to dementia [269].

## 9. Conclusions

Our exponentially improving diagnostic possibilities have enabled us to identify AD at an early stage and proved that brainstem suffers the earliest loss during the disease. Therapeutic tools, however, do not parallel our spectacular progress in diagnosis. This is particularly due to our poor understanding of the complex molecular mechanisms which trigger AD. Progress has been made exploring the effects of life style interventions [270], and efforts to improve pharmacotherapy are being continued [271]. We believe that exploring brainstem pathology in select neuronal subsets will not only help the early recognition of this hitherto incurable neurodegenerative illness but offer promising possibilities for future therapy.
